# Outcomes of patients with bacteriuria/pyuria of clinically undetermined significance (BPCUS) treated with antibiotics in 23 community hospital emergency departments

**DOI:** 10.1017/ash.2023.204

**Published:** 2023-06-30

**Authors:** John J. Veillette, C. Dustin Waters, Jared Olson, George Vargyas, Emily M. Ingalls, Mary A. Hutton, Nick Tinker, Stephanie S. May, Rachel A. Foster, Jena Stallsmith, Todd J. Vento

**Affiliations:** 1 Infectious Diseases Telehealth Service, Intermountain Healthcare, Murray, UT, USA; 2 Department of Pharmacy, Intermountain Medical Center, Murray, UT, USA; 3 Department of Pharmacy, McKay-Dee Hospital, Ogden, UT, USA; 4 Department of Pharmacy, Primary Children’s Hospital, Salt Lake City, UT, USA; 5 Division of Infectious Diseases, Department of Pediatrics, University of Utah, Salt Lake City, UT, USA; 6 Utah Emergency Physicians, Intermountain Medical Center Emergency Department, Murray, UT, USA; 7 Department of Pharmacy, Utah Valley Medical Center, Provo, UT, USA; 8 Division of Clinical Epidemiology and Infectious Diseases, Intermountain Medical Center, Murray, UT, USA

## Abstract

The optimal management of bacteriuria/pyuria of clinically undetermined significance (BPCUS) is unknown. Among 220 emergency department patients prescribed antibiotics for BPCUS, we found frequent readmissions, which were mitigated by outpatient follow-up visits. Observation and follow-up for an unknown diagnosis should be emphasized over antibiotics due to high likelihood of readmissions.

## Background

Many clinical syndromes are attributed to “urinary tract infection” (UTI) in the emergency department (ED) due to excessive urine testing and the prevalence of bacteriuria.^1–3^ One challenging scenario involves patients lacking UTI symptoms or systemic signs of infection but presenting with bacteriuria plus non-localizing symptoms of unclear etiology (eg, abdominal pain, weakness, and altered mental status). This syndrome has recently been labeled bacteriuria/pyuria of clinically undetermined significance (BPCUS); however, unlike asymptomatic bacteriuria (ASB) and cystitis, the prevalence and optimal management of BPCUS is unknown.^1,4,5^ Therefore, the purpose of this study was to describe the management and outcomes of BPCUS compared to ASB or cystitis.

## Methods

We performed two cross-sectional evaluations^6,7^ of antibiotic prescribing for UTI in 23 EDs in Utah and Idaho (14 to 503-bed hospitals; 9 rural, 14 urban) in the Intermountain Healthcare system, an integrated not-for-profit network of hospitals and clinics. Briefly, these studies included adult patients with an ED visit in 2017 or 2021 who had a urinalysis (UA) ordered, were diagnosed with “UTI” (identified via ICD10 code), and were discharged with an antibiotic prescription. Patients with a concomitant infection, receiving antibiotics prior to ED visit, neurogenic bladder, pregnancy, or pyelonephritis were excluded. UA results, antibiotic prescriptions, demographics, chief complaint, and 14-day ED readmissions were extracted electronically. ED notes were reviewed by an infectious diseases (ID) pharmacist using a standardized data collection form (Supplemental Material) to determine a diagnosis (cystitis, ASB, or unknown) and whether ED readmissions were attributed to UTI by the ED provider. Charts were further reviewed to capture follow-up primary care visits within 14 days, which often took place within our network.

Patients with focal UTI symptoms (dysuria, urgency, frequency, or suprapubic pain) were classified as cystitis, while those with an abnormal UA (positive leukocyte esterase, positive nitrites, ≥5 WBCs, or presence of bacteria) lacking UTI symptoms were classified as ASB. UA was used in lieu of urine culture to define ASB because ED prescribing is influenced by UA findings and culture results are not available during the ED visit. Patients with an abnormal UA and only non-localizing symptoms were classified as ASB if the ED provider documented a clear non-UTI diagnosis as the cause (eg, abdominal pain caused by constipation), but otherwise were classified as unknown. Of those with an unknown diagnosis, we further excluded patients with systemic signs of infection (eg, fever, leukocytosis) leaving only patients who met the criteria for BPCUS. Validation of 8% of all cases and 25% of BPCUS cases by a second ID pharmacist yielded an interrater agreement of 91% and a kappa value of 0.82 (95% CI 0.71–0.93) for the classification of BPCUS.

The primary endpoint was 14-day all-cause readmissions. Secondary endpoints included UTI-related readmissions and the percent of patients receiving antibiotic durations >7 days or a fluoroquinolone. All readmissions were reviewed by the same ID pharmacist. Fisher’s exact test was used for pairwise comparisons of categorical data, and *P* values <0.05 were considered statistically significant. The study was exempted by our Institutional Review Board as a quality improvement project.

## Results

**Figure 1.**
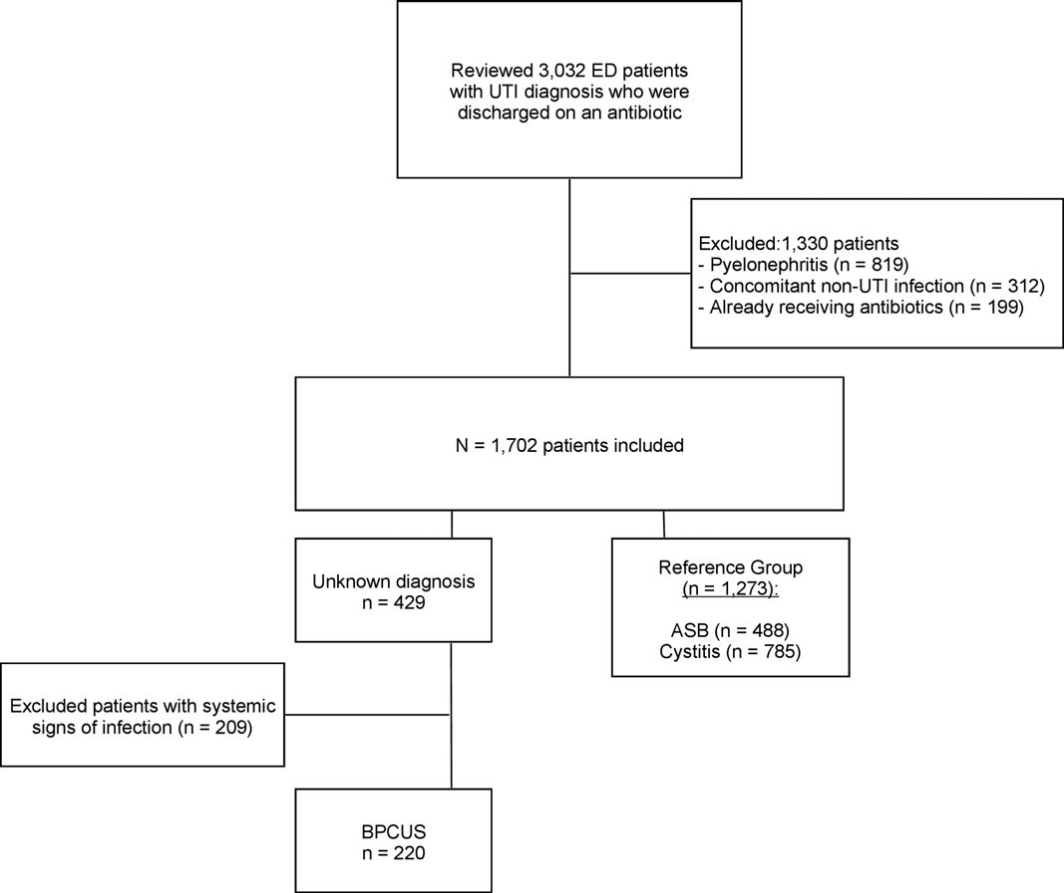
Patient flowchart. ASB, asymptomatic bacteriuria; BPCUS, bacteriuria/pyuria of clinically undetermined significance; ED, emergency department; UTI, urinary tract infection; Systemic signs of infection: fever, leukocytosis, hemodynamic instability.

Of 3,032 patients reviewed, 1,702 (56%) were included: 1,273 (42%) had ASB or cystitis, 429 (14%) had an unknown diagnosis, and 220 (7%) met criteria for BPCUS (Fig. 1). A similar number of patients were male in the BPCUS, ASB, and cystitis groups, but more BPCUS patients were ≥80 years of age (Table 1). The most common chief complaints among the 220 BPCUS patients were abdominal pain (n = 65, 30%), flank/back pain (n = 45, 20%), weakness/dizziness (n = 39, 18%), and altered mental status (n = 33, 15%).

**Table 1.** Demographics, antibiotic treatment, and outcomes

	BPCUS (n = 220)	ASB (n = 488)	*P* value^*^	Cystitis (n = 785)	*P* value^**^
**Demographics**					
Age ≥ 65 years	110 (50)	223 (46)	0.292	322 (41)	**0.021**
Age ≥ 80 years	56 (25)	101 (21)	0.172	116 (15)	**<0.001**
Male	45 (20)	86 (18)	0.403	139 (18)	0.375
**Antibiotic treatment**					
Duration >7 days	56 (25)	105 (22)	0.247	167 (21)	0.199
Treated with FQ	42 (19)	95 (19)	1.000	169 (22)	0.455
**Outcomes**					
14-day all-cause readmission	50 (23)	85 (17)	0.099	117 (15)	**0.008**
14-day UTI-related readmission	7 (3)	4 (1)	**0.041**	47 (6)	0.127

Data are listed as number (percent); ASB, asymptomatic bacteriuria; BPCUS, bacteriuria/pyuria of clinically undetermined significance; FQ, fluoroquinolone; UTI, urinary tract infection.

* Compares BPCUS versus ASB.

** Compares BPCUS versus Cystitis.

Fourteen-day all-cause ED readmissions were higher with BPCUS than ASB or cystitis (Table 1). Of the 50 BPCUS readmissions, 28 (56%) presented with similar symptoms to their index ED visit (Table S1, Supplemental Material). The median time to ED readmission was 4.5 days (IQR 2.0–9.7 days). The likelihood of readmission was significantly lower among BPCUS patients who had a follow-up visit with their primary care provider within 14 days of the index ED visit compared to those who did not [2/55 (4%) versus 48/165 (29%), respectively, *P* < 0.001].

UTI-related readmissions were more likely with BPCUS than with ASB and less likely with BPCUS than with cystitis although the difference between BPCUS/cystitis was not statistically significant (Table 1). Regarding the 7 UTI-related readmissions among BPCUS patients, 2 (29%) were diagnosed with pyelonephritis, 3 (43%) were diagnosed with cystitis, and 2 (29%) had abdominal pain that was attributed to UTI. There were no significant differences between groups for antibiotic duration >7 days or fluoroquinolone use (Table 1). Among included patients, a total of 1,570 days of antibiotics were prescribed for BPCUS compared to 3,394 days for ASB and 5,433 days for cystitis (including 528 excess days from cystitis courses >7 days).

## Discussion

Overtreatment of UTIs in the ED is an important target for antibiotic stewardship programs (ASPs). Targeting excessive prescribing for cystitis and ASB is a logical first step due to higher volume and the availability of evidence-based guidelines to define appropriate management.^1,4^ However, our data suggest that decreasing antibiotic prescribing for BPCUS might represent an additional opportunity since this syndrome accounted for 7% of patients prescribed antibiotics for UTI in the ED.

While the optimal management of BPCUS is unknown, experts suggest that supportive care, observation, and follow-up should be emphasized, while urine testing and antibiotic treatment should be avoided in the absence of sepsis.^1,8,9^ This is especially true for elderly patients, who have a higher risk of ED readmission^10^ and accounted for 25% of our BPCUS cases. Our data support IDSA guideline recommendations^1^ for elderly patients with ASB plus altered mental status or functional decline: observation and assessment for other causes should be emphasized over antibiotic treatment (although further study is needed in this area). The importance of follow-up was supported by our findings that having a follow-up primary care visit within 14 days had a protective effect against readmission.

Our study has several limitations. Under-reporting of symptoms may have confounded the identification of BPCUS (versus ASB or cystitis) on retrospective chart review, reviewers were not blinded to the study objectives, and healthcare encounters outside of our system were not captured. We did not capture urine culture results, changes in antibiotics after discharge, or other outpatient encounters. We also did not perform a multivariate analysis to identify risk factors for ED readmission. Most notably, we only identified patients who were treated with antibiotics; thus, we were unable to compare outcomes of BPCUS treatment versus non-treatment. The nature of uncertainty creates an environment of not wanting to miss something, and it is possible that some BPCUS patients might benefit from antibiotics. However, the high rate of readmissions despite antibiotic treatment suggests that ED providers should emphasize close follow-up (if feasible) for an unknown diagnosis and avoid anchoring on an abnormal UA to guide treatment.

In summary, BPCUS accounted for a sizeable portion of ED antibiotic prescribing for UTI, although it was less common than ASB, cystitis, or pyelonephritis. While ED providers are hesitant to call BPCUS patients “asymptomatic,” it is unclear if antibiotic treatment offers any benefit. Supportive care, observation, and close follow-up for an unknown diagnosis should be emphasized over treatment with antibiotics due to the high likelihood of ED readmissions.

## Supplementary material

For supplementary material accompanying this paper visit http://doi.org/10.1017/ash.2023.204. click here to view supplementary material
