# Supplementary material for: Outcomes of patients with bacteriuria/pyuria of clinically undetermined significance (BPCUS) treated with antibiotics in 23 community hospital emergency departments
Source: Antimicrob Steward Healthc Epidemiol. 2023 Jun 30;3(1):e114. doi: 10.1017/ash.2023.204 (PMC10369435; doi:10.1017/ash.2023.204)
Supplement: Supplementary file 1 [file S2732494X23002048sup001.pdf]

Table S1. Final diagnoses of 28 BPCUS patients with similar chief complaints in 2 consecutive ED visits

Chief complaint for both ED visits	*Final diagnoses after 2 ED visits*
Abdominal pain (n = 8)	Unknown cause for symptoms (n = 7)<br>Cholecystitis (n = 1)
Flank pain (n = 7)	Kidney stone (n = 4)<br>Musculoskeletal pain (n = 2)<br>Leaking nephrostomy tube (n = 1)
Weakness/dizziness (n = 7)	Unknown cause for symptoms (n = 1)<br>Mechanical fall (n = 1)<br>Prostate cancer (n = 1)<br>Heart failure exacerbation (n = 1)<br>Hyponatremia (n = 1)<br>Spinal stenosis (n = 1)<br>*Staphylococcus aureus* bacteremia + vertebral osteomyelitis (n = 1)
Altered mental status (n = 3)	Unknown cause for symptoms (n = 1)<br>Dehydration (n = 1)<br>Psychosis (n = 1)
Urinary retention (n = 1)	Benign prostatic hyperplasia with obstruction (n = 1)
Chest pain/epigastric pain (n = 1)	Unknown cause for symptoms (n = 1)
Seizure (n = 1)	Unknown cause for symptoms (n = 1)
